# Exploring the Association between Welfare State and Mental Wellbeing in Europe: Does Age Matter?

**DOI:** 10.3390/ijerph191710985

**Published:** 2022-09-02

**Authors:** Jorid Kalseth, Valeria Donisi, Marta Miret, Anna K. Forsman, Johanna Cresswell-Smith

**Affiliations:** 1 Department of Health Research, SINTEF Digital, Pb. 4760 Torgarden, 7465 Trondheim, Norway; 2 Department of Neurosciences, Biomedicine and Movement Sciences, University of Verona, 37134 Verona, Italy; 3 Centro de Investigación Biomédica en Red de Salud Mental (CIBERSAM), Department of Psychiatry, Universidad Autónoma de Madrid, 28029 Madrid, Spain; 4 Health Sciences, Faculty of Education and Welfare Studies, Åbo Akademi University, 65101 Vaasa, Finland; 5 Mental Health Unit, Finnish Institute for Health and Welfare, 00271 Helsinki, Finland

**Keywords:** subjective wellbeing, psychological wellbeing, mental wellbeing, age gradient, welfare state

## Abstract

Previous research reports show mixed results regarding the age gradient in population mental wellbeing, which may be linked to the role that welfare states play. In this study, we investigate whether an age gradient exists in relation to the association between welfare state and mental wellbeing within the adult population in Europe. We combine individual level data from Round 6 of the European Social Survey and country level data on welfare state and use multilevel regression analyses to explore population mental wellbeing. Subjective and psychological wellbeing dimensions were analyzed, and different approaches to measuring welfare state were explored, including a regime typology and composite welfare state measures constructed on the basis of a set of eight individual indicators. We found the age gradient for mental wellbeing to differ between welfare states, with the positive impact of the welfare state increasing with age. A universal and generous welfare state seems to be particularly important for older adults, who are also more likely to be in higher need of transfers and services provided by the welfare state.

## 1. Introduction

### 1.1. Age, Mental Wellbeing, and the Welfare State

The share of adults aged 80 years and over, often referred to as the oldest old, is projected to more than double in Europe by 2050 [1]. This demographic transition has spurred increased focus on policies supporting good levels of health and wellbeing in older age. The United Nations has declared the 2020s as the Decade of Healthy Ageing, defined by the World Health Organization as “the process of developing and maintaining the functional ability that enables wellbeing in older age”. Healthy ageing is therefore recognized as much more than absence of disease, and functional ability is defined as being able to do whatever feels valuable.

Health outcomes and mental wellbeing (MWB) are closely linked in older age, making maintaining these a special area of interest [2]. Healthy ageing can be seen to result from the interaction of intrinsic individual capacities and environmental or context characteristics [3]. Strong national health-in-all policies supporting individual autonomy and participation and sustainable health systems (including equitable long-term care systems) aligned to the needs of the older population are key strategies for healthy ageing [4].

Welfare state (WS) refers to a type of governance in which national governments secure the basic wellbeing of all citizens, for example by granting and protecting social rights, or by providing security and equality to their citizens [5]. The hallmark of a strong WS is comprehensive welfare policies, which include all levels and sectors of government. WS may impact health and wellbeing via policies for promoting capacity-enhancing behaviors, removing barriers to participation, and compensating for loss of functional capacity [3]. Reducing market reliance may also be particularly important for the wellbeing of people with substantial care needs. Although functional capacity is heterogeneous in older ages, ageing often entails increased support needs, making WS policies especially important to this age group [3].

### 1.2. Mental Wellbeing over the Life Course

In recent years, the literature base on how broader determinants of population health [6] influence MWB [7] has grown. MWB is associated with good psychosocial functioning, better physical health, lower health care utilization, less work absenteeism [8], and has been described as a determinant of healthy ageing [2,9]. High levels of MWB can therefore be assumed to be beneficial on both the individual level and societal level.

MWB is a multifaceted concept [10,11]. Although terminology is far from consistent, mental wellbeing as a concept tends to center around different dimensions capturing aspects from two dominant research traditions. One tradition links wellbeing to happiness and life satisfaction and is often associated with the term subjective wellbeing (SWB) [12]. SWB covers both life-evaluation, i.e., cognitive appraisal of overall happiness and satisfaction in life (also called evaluative wellbeing), and hedonic experience, i.e., affective/emotional states of life satisfaction (also called emotional wellbeing). Another leading tradition places greater emphasis on personal development and self-realization, reflecting positive functioning and personal expressiveness including purpose, mastery and positive relatedness, flow, autonomy, personal growth, and self-acceptance. This latter approach is often associated with the term psychological wellbeing (PWB) [13].

The way in which these MWB approaches explain patterns of change over the life course vary [14]. Ulloa et al. [15] discuss wellbeing over the life cycle from different perspectives, including the field of economics, psychology, and gerontology, many of which were found to be consistent with a so-called flat wellbeing curve, e.g., theories of age-independent utility and consumption smoothing, expectation adaption to fixed aspiration levels, or level of happiness being determined by genes and personality. According to these theories, wellbeing responses to changes in circumstances are only transitory. However, long-term effects have also been reported in the empirical literature, for example in response to major life events, such as changes in family situation, health, and labor market participation (ibid.). In old age, these changes may relate to reduced independence and increased social isolation (e.g., decline in physical and mental health, loss of friends and loved ones, as well as work-related social contacts, etc.), which have been found to be negatively related to MWB [16,17]. Major life events associated with old age could therefore be assumed to result in a negative age gradient in relation to wellbeing.

However, several studies have found life satisfaction and happiness to be higher in older age compared to middle age. Although the existing literature remains inconclusive, evaluative wellbeing, or life satisfaction, has been found to follow a U-shaped curve across the life course, with a mid-life nadir, i.e., the lowest level of wellbeing occurring around 50 years of age [18,19]. This finding is consistent with socio-emotional selectivity theory [20], which proposes that older adults tend to focus more on the present and on things that generate a sense of wellbeing, with less emphasis on other things that may pay off in the future [15]. Furthermore, although older adults may have fewer social contacts, they have been found to be more meaningful, which has the potential to reduce emotional stress and improve wellbeing [21]. These findings imply that wellbeing can be maintained into older age via adaptation to age-related change. However, insights from the field of gerontology suggest that the ability to adapt declines in oldest old age [15], which has the potential for negative impacts on MWB in this age group compared to younger older adults.

The theories and empirical findings outlined above refer to general age patterns of life satisfaction and happiness. At the individual level, the age pattern may vary, reflecting different individual circumstances such as health, family and living situation, income and wealth, and other major life events people experience over the lifespan. Furthermore, the U-shaped age pattern for wellbeing is not a universal observation [15] and has been shown to vary regionally [2], pointing to potential WS influences on MWB. WS may influence MWB by impacting individual circumstances, but also via its impact on the environment surrounding the individual. Alesina et al. [22], for example, found it less likely that individuals report themselves as happy in societies with higher levels of inequality, even after controlling for individual income and other personal characteristics. The strength of both mechanisms may vary according to age and hence contribute to the age gradient in MWB to differ between WS.

### 1.3. Welfare State Impact on Health and Wellbeing

Previous research has found social democratic WS regimes to present the most favorable results in terms of absolute health outcomes [23]. The Nordic WS, with strong redistributive and universal welfare policies [24], has been found to perform well on various health and wellbeing outcomes such as self-perceived health [25], adolescent health [26], infant mortality rate and birth weight [27], oral health [28], employment-related health [29], as well as subjective wellbeing [30,31]. Comprehensive WSs seem to contribute to increased happiness and life satisfaction [32]. For example, Deeming and Jones [33] found self-perceived health and subjective wellbeing (specifically happiness and life satisfaction) to be linked to European WS regimes, with Nordic countries displaying most favorable outcomes, and Post-Communist countries the least favorable outcomes. Furthermore, the Great Recession of the late 2000s was associated with lower scores in happiness and life satisfaction outcomes for Anglo-Saxon countries, while Post-Communist countries showed improvement in these outcomes, and Nordic countries showed minimal changes [33].

WSs support health and wellbeing by redistributing resources via two key mechanisms [34]. The so-called “Robin Hood function” involves reducing social exclusion and redistributes resources from those who have a lot to those that have less (e.g., via income taxation). The second mechanism, the so-called “Piggy Bank function” insures against social risks by redistributing resources over the life course, that is, from productive to unproductive stages (e.g., via insurance and pension systems). How these two mechanisms influence health and wellbeing over the life course is an interesting area of investigation, in particular, whether WS effect on MWB differs over the life course and is most influential during less productive ages.

### 1.4. Study Objectives and Research Questions

The current study tries to bridge two branches of literature, namely literature on the impact of WS on health and wellbeing and the literature on the age gradient of MWB. Ultimately, the aim is to explore whether there is an age gradient in the WS impact on MWB. To the best of our knowledge, the age gradient of WS influence on MWB has not previously been explored in a European setting. The current study therefore aims:

(i) to explore the association between MWB and WS;
(ii) to explore the age gradient in the association between MWB and WS.

The current study builds on findings from the EMMY project, an interdisciplinary and mixed methods comparative study on the impact of welfare systems on MWB among the oldest old. Construct measures of MWB were built using individual level data from the sixth round of the European Social Survey (ESS). Different approaches to measuring welfare state were explored, including regime typology and composite welfare state measures. The results of our study highlight the importance of welfare state policies for the mental wellbeing of older people in particular.

## 2. Materials and Methods

### 2.1. Data Materials

Analyses included data from 24 countries belonging to the European Union (EU) or the European Free Trade Association (EFTA) who participated in Round 6 of the European Social Survey (ESS) [35]. The MWB measures were constructed using individual level data obtained from the ESS data. This data set was chosen due to its broad cover of items measuring different aspects of MWB. The ESS includes data from individuals aged 15 years and older, although we have limited our analyses to the adult population (18+), with no upper age limit.

Data on various country level WS indicators were retrieved from different open data sources, including Eurostat, the World Bank DataBank, ESS Multilevel Data Repository and Hofstede Insights (see Table A2 for more details). Exploratory Factor Analysis was used to construct the composite WS indices (as described in Section 2.3), based on data from all EU or EFTA countries with non-missing data on the relevant WS indicators.

### 2.2. Measuring Mental Wellbeing

Our approach builds on a previous analysis outlining MWB dimensions using exploratory structural equation modelling (ESEM) on the same ESS data set [36]. The analysis applied a positively slanted approach [10,11] also reflected in Seligman’s PERMA model [37], the Warwick–Edinburgh Mental Well-Being Scale (WEMWBS) [38] as well as the WHO-5 Well-Being Index [39]. As reported in our previous study [36], MWB may be represented by a six-factor model comprising “evaluative wellbeing”, “positive emotional wellbeing”, “positive functioning”, “flow”, “positive relationships”, and “social engagement”. Social wellbeing, captured by the social engagement factor, is typically treated as separately from SWB and PWB [40,41]. This factor was also found to result in low internal consistency and scale reliability in our previous analysis [36] and was therefore excluded in the present analyses.

For the analyses to be more manageable, we merged the five retained factors into two MWB dimensions representing SWB and PWB. SWB and PWB were constructed in a three-step procedure as follows:

- All individual items (*X*) were normalized (*Xnorm*) using the min–max algorithm such that all items take on values in the interval 1–5:
  *Xnorm* = ((5 − 1)/(*Xmax* − *Xmin*)) × (*X* − *Xmax*) + 5
- Each of the five retained MWB factors from the original ESEM analysis were calculated as means of the normalized items included. See Table A3 for an overview of items included in each of the five factors.
- SWB was measured as the mean of “evaluative wellbeing” and “emotional wellbeing” factors, whereas PWB was measured as the mean of “positive psychological functioning”, “flow”, and “positive relationships” factors.

### 2.3. Welfare State Measures

Three operationalizations of WS were used, one based on regime typology and two based on a composite index approach using factor analysis to produce WS dimensions. Approaches to measure WS are elaborated in Appendix A. The following paragraphs describe the construction of WS variables in more detail.

#### 2.3.1. Regime Typology (Dummy Variable Approach)

The 24 countries were grouped into the following five WS regime types often used for the European context [25,33]: (i) Nordic/Social Democratic (including Denmark, Finland, Iceland, Norway and Sweden), (ii) Bismarckian/Conservative (including Belgium, France, Germany, the Netherlands, Switzerland), (iii) Anglo-Saxon/Liberal (including United Kingdom, Ireland), (iv) Southern/Mediterranean (including Cyprus, Italy, Portugal, Spain), (v) Eastern/Post-Communist (including Bulgaria, Czech republic, Estonia, Hungary, Lithuania, Poland, Slovakia, Slovenia). See Appendix A for elaborations.

#### 2.3.2. Welfare State Index

There are many potential WS indicators of relevance to MWB measuring specific aspects of WS such as welfare culture, welfare institutions and policy instruments. In his seminal work on WS regimes, Esping Andersen [42] identified several areas of WS variation such as social rights and social spending, income redistribution, and employment policy. The human value and civil rights aspect of welfare culture, with its socio-structural effects highlighted in the ideal-types proposed by Rice [43] (cf. Appendix A), points to value-oriented variables such as individualism, social trust and gender (in)equality [24,32,44]. Including gender inequality into the WS formulation also responds to the “neglect of gender-perspective” critique of the Esping Andersen tradition [45,46]. Furthermore, a review by Jorm and Ryan [47] found that subjective wellbeing was associated with income per capita, income inequality, social welfare, individualism, democracy and freedom, social capital and physical health. Our list of indicators, all measured at the national level, were based on these findings, comprising eight variables covering aspects of WS, including income level, service and benefits generosity, gender inequality, longevity, labor market characteristics, income inequality, individualism, and societal trust (Table A2).

In order to construct one composite WS index while allowing for different weights of the WS indicators, factor analysis was used to restrict the number of factors into one. The predicted value for this factor was used to form the WS index (see Table A4).

#### 2.3.3. Welfare State Factors

Using an unrestricted factor analysis applying the Kaiser criterion (eigenvalue >=1 using the principal component factor method), two uncorrelated WS factors were found (see Table A4). The first factor (WS factor 1) includes high loading for service and benefits generosity, gender equality and longevity. The second factor (WS factor 2) captures societal trust, as well as individualism, working life duration, and income inequality. Income level, however, resulted in the same loading on both factors. The predicted values of the two WS factors are used as variables in regression analyses.

The use of factor analyses to form the WS dimensions/factors allowed the potential correlation between different WS indicators (separate variables, cf. single indicator approach described in Appendix A) to be accounted for, while also allowing for different weightings of the individual indicators.

### 2.4. Statistical Analyses

The association between MWB (represented by two approaches i.e., SWB and PWB) and WS was analyzed using the following three models; the WS index (Model 1), WS factors (Model 2) and WS regime typology (Model 3). The analyses were performed using random intercept multilevel regression models, with individuals nested within countries, and with weighting adjustments based on post-stratification weights [48]. The use of a multilevel regression model allowed us to consider within-country correlation in the random error component. For reference, we used a model without country-level WS variable(s) (Model 0). The intraclass correlation coefficient (ICC) was calculated to describe the proportion of total variation observed at the country level. The different models were evaluated by the Akaike information criterion (AIC) and Bayesian information criterion (BIC), as well as by comparing explained variance (R2) at the country level using the methods of Bryk and Raudenbush [49] and Snijders and Bosker [50]. We also performed analyses for each of the eight WS indicators (used to construct the WS index and WS factors) separately for comparison. The results for R2 at the country level are documented in Table A5.

A dummy-variable specification of age including dummies for seven age groups (i.e., 18–29, 30–39, 40–49, 50–59, 60–69, 70–79, 80+) were used to allow for non-linearity in the age gradient. Moreover, a test for an age gradient in WS impact was performed by including interaction variables of age dummies and WS. All analyses controlled for gender.

The aim of the study was to investigate the overall association between WS and the age gradient in MWB. The association between MWB and WS could potentially be mediated by individual level characteristics. Mediation implies that the effect of WS on MWB can (partly) be related to WS influencing individual level characteristics (e.g., income), which in turn influences MWB. Hence, controlling for individual level variables in the analysis is likely to reduce the magnitude of the WS association with MWB, potentially masking the WS effect on the age gradient in MWB. Therefore, our analyses do not include other individual level variables than age and gender.

**Figure A1:**
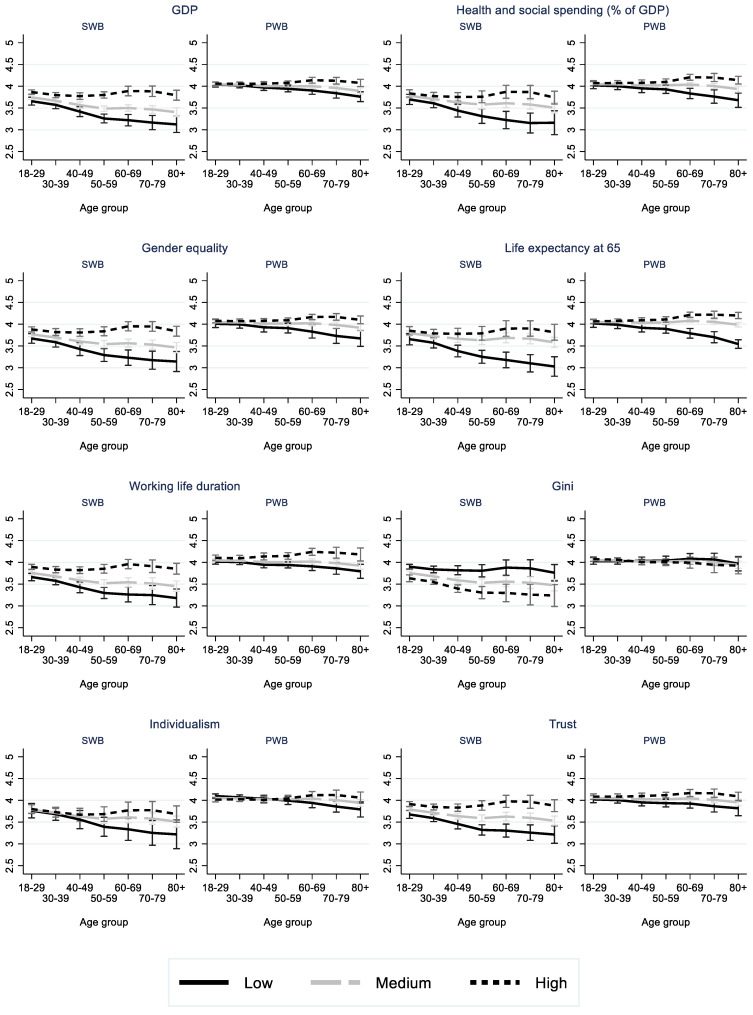
Predicted level of subjective wellbeing (**SWB**) and psychological wellbeing (**PWB**) by age and high (p90), medium (p50) and low (p10) values of individual WS indicators. Results from multi-level regressions. NB Scale in Figure 2.5–5.

Predicted values of SWB and PWB for different values of WS models are illustrated graphically. When calculating predictions based on the results for WS factors and WS index approaches, low (10th percentile (p10), i.e., third lowest), median and high (90th percentile (p90), i.e., the third highest) value of the WS factors/index were used. Figure A1 shows predicted values of SWB and PWB from the analyses using the individual WS indicators as WS measure.

## 3. Results

### 3.1. Values of WS Index and WS Factors by WS Regime Types

**Figure 1:**
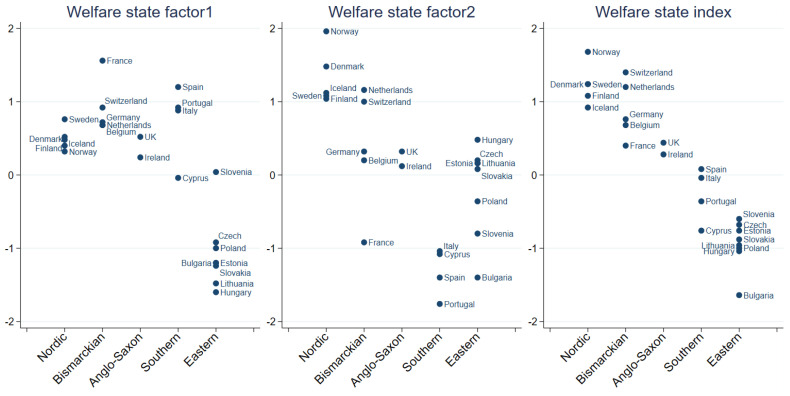
Country score on WS Factors and WS Index by WS regime type.

Figure 1 shows the relation between WS regime types and the WS factors, as well as the WS index for the 24 countries included in the MWB regression analyses.

WS factor 1 (with a few exceptions) separates the Eastern European countries from the rest. Furthermore, the Nordic countries can be seen to score on the higher side on WS factor 2, with Bismarckian (except for France) and Anglo-Saxon countries scoring close to or above average, and Eastern European countries scoring close to, or below average, while the Southern countries scored below average. When the different aspects of WS were expressed by a composite WS index, a clear pattern emerged. Average values were highest in the Nordic countries, followed by the Bismarckian countries, the Anglo-Saxon countries, followed by Southern countries, and finally the Eastern European countries which held the lowest average value. This approach appears to confirm that WS variations are clustered by regime types to a high degree. The correlation matrix for the composite WS variables and the individual WS indicators used to construct the variables are shown in Table A6.

### 3.2. Descriptive Statistics

After excluding observations with missing data on age and/or gender variables, the current analysis included a sample of 43,552 individuals, aged 18 year or older (Table 1). The regime type split in the table was also chosen due to its representation of geographical regions in Europe.

The mean score is significantly lower, and the variation higher, for the SWB measure in comparison to the PWB measure across all regime types.

The data for Nordic and the Bismarckian groups are more gender-balanced than the other regime types, which have a clear majority of female respondents (±55 percent women). The age distribution, however, is relatively similar between WS regime types, with the mean age (not shown) being around 50 years of age in all regime types.

### 3.3. Multivariate Regression Results

**Figure 2:**
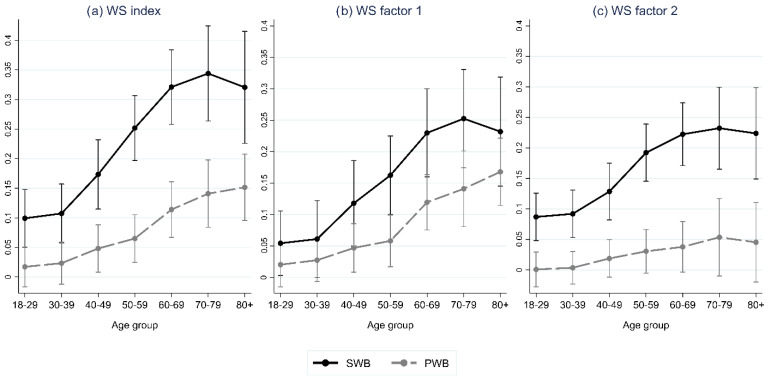
Results from multilevel regression of subjective wellbeing (SWB) and psychological wellbeing (PWB). Marginal effects of WS index from Model 1 (**a**), WS factor 1 (**b**) and WS factor 2 (**c**) from Model 2, by age group.

**Figure 3:**
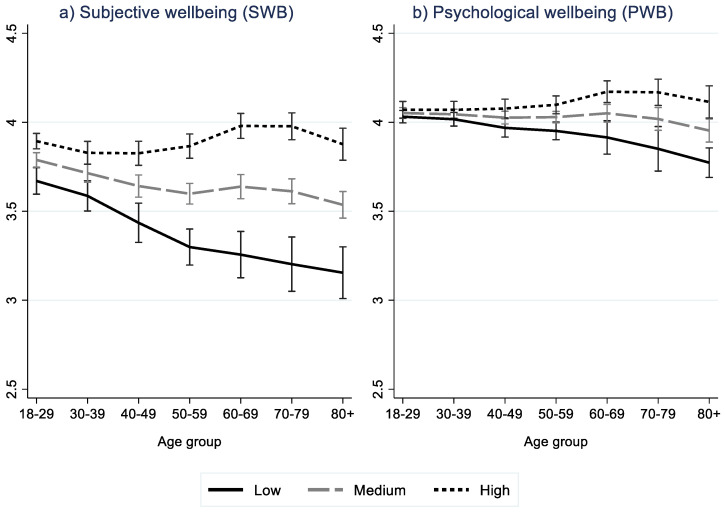
Predicted level of subjective wellbeing (**a**) and psychological wellbeing (**b**) by age and high (p90), medium (p50) and low (p10) value of WS index. Results from multilevel regression (Model 1). NB Scale in Figure 2.5–4.5.

**Figure 4:**
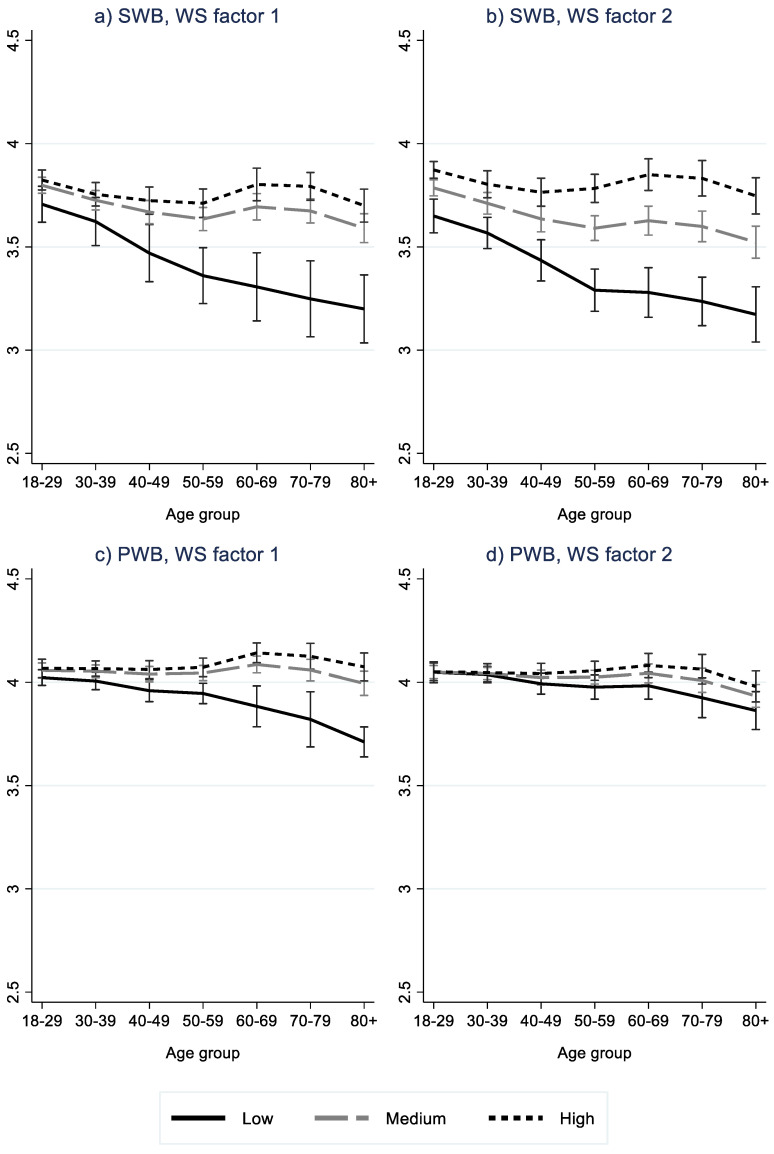
Predicted level of subjective wellbeing (**a**,**b**) and psychological wellbeing (**c**,**d**) by age and high (p90), medium (p50) and low (p10) values of WS Factor 1 (**a**,**c**) and WS Factor 2 (**b**,**d**). Results from multi-level regression (Model 2). NB Scale in Figure 2.5–4.5.

**Figure 5:**
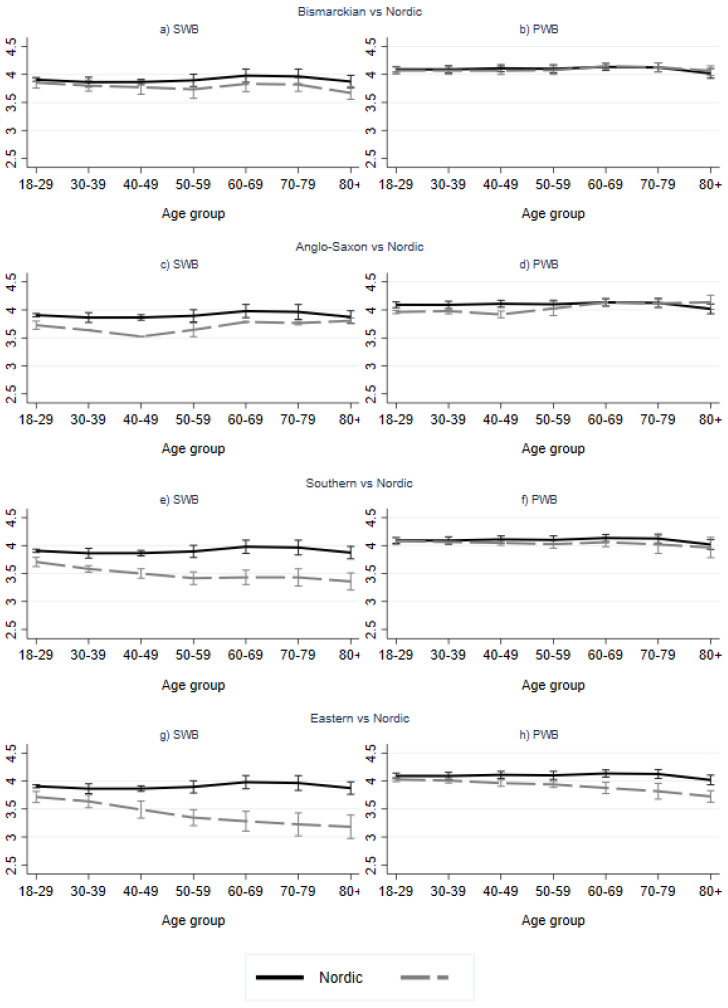
Predicted level of subjective wellbeing (**a**,**c**,**e**,**g**) and psychological wellbeing (**b**,**d**,**f**,**h**) by age and WS regime type (Bismarckian (**a**,**b**), Anglo-Saxon (**c**,**d**), Southern (**e**,**f**) and Eastern (**g**,**h**)). Nordic is reference group. Results from multilevel regression (Model 3). NB Scale in Figure 2.5–4.5.

Results of multilevel regression analysis are shown in Table 2. Marginal effects of WS index and WS factors, by age group, are shown graphically in Figure 2, while SWB and PWB predictions by age group and WS are shown in Figure 3, Figure 4 and Figure 5. The ICC for the model without WS variable(s) shows that ten percent of the total variation in SWB was at the country level. The corresponding for PWB was lower, at 4.5 percent.

Differences in SWB and PWB between age groups emerged. For Europeans on average, i.e., model 0, SWB decreases with age until 50–59 years, and then, there is a weak increase for the 60–69 group and a decrease for the older groups. The SWB for the 80+ years group was significantly lower than for other age groups, except for 50–59 years. Lowest levels for the oldest old (80+ years) emerged also for PWB, significantly lower than all other age groups.

Overall, the estimates of the WS variables and the country-level R2 show that the WS effect varies by age group and was stronger for the SWB measure of MWB in comparison to PWB. The ICC after inclusion of WS variable(s), i.e., remaining variation at the country level, also drops more (both absolutely and relatively) for SWB than PWC.

The results indicate that level of WS in terms of higher values of the WS index, is associated with higher levels of SWB and PWB. The estimates of the WS index show a moderate positive effect for SWB and no effect for PWB among the young adult groups (<40 years). The marginal effect of the WS index increased with age until old age (70+ years).

The direction of the age gradient varied both with age and WS index level. A negative age gradient was found for low levels of the WS index, while a positive age effect was found for high values of WS index, however only among the middle-aged group (between 40/50 and 60 years).

Both WS factors showed similar results as the WS index for SWB. For PWB, only WS factor 1 resembles the results of WS index, i.e., a significant effect (among 40+ years), which increases with age. The second WS factor was found not to be significantly associated with PWB for any age group.

In terms of WS regime, results show less favorable MWB (both SWB and PWB) scores for the Eastern European regime type compared to the Nordic regime type (used as the reference category), especially in higher age groups. Furthermore, lower levels of SWB were found in the Southern European region compared to the Nordic, this difference increasing into older age (≥60). Likewise, we found lower levels of SWB in the Anglo-Saxon region compared to the Nordic region, with largest differences found for the 40–49 age group, and insignificant differences for the oldest old age group (≥80). PWB was also found to be higher in the Nordic regime type compared to the Anglo-Saxon type for the under 50 age groups, this difference being highest for the 40–49 age group. The difference between these two regime types was non-significant for the 50–79 age group, but positive in favor of the Anglo-Saxon regime vs. the Nordic for the oldest old age group (≥80). No differences were found between the Nordic and the Bismarkian regime, apart from a lower SWB score for the oldest old age group in the Bismarckian regime.

In summary, a largely similar age pattern was found for the Nordic and Bismarckian regimes with a tendency for SWB to decrease from a younger to middle age, and then increase toward the ages of 60–79 to then decrease again in oldest old age (significantly lower than all other age groups for the Bismarckian regime). For PWB, highest values were found among the 60–79-year-olds, with lower values for the oldest old age group (this value was significantly lower than all other age groups in the Nordic region). For the Anglo-Saxon regime, we found the lowest values for both SWB and PWB in the 40–49 age group and higher values for older age groups. The Southern regime resulted in a negative age effect for SWB up to the ages of 50–79, and a further decrease in oldest old age. Moreover, we found significantly higher scores for PWB in the 60–69 age group compared to the 50–59 group, as well as for the 70–79 age group in comparison to the over 80 age group. For the Eastern region, a negative age gradient was found for both SWB and PWB.

According to the Snjider/Bosker and Bryk/Raudenbush R-squared regarding country level variation, the WS index and WS factors approaches were found to explain the variation in SWB to a greater extent compared to the WS regime approach. The opposite was found for PWB. Notably, the BIC was lowest for the WS index and WS factor approaches, while AIC was lowest for the WS regimes approach.

Most of the eight individual WS indicators explained the variation of SWB and PWB to a lesser extent compared to the composite WS measures (see Table A5). However, GDP per capita was found to have a higher country-level R-squared for SWB in comparison to the WS regime typology, whereas longevity explained the same amount of variation in PWB as the WS index. The country-level R-squared for the Gini coefficient and individualism for PWB are close to zero. The age gradient of predicted SWB and PWB for the eight WS indicators correspond, even though to a varying degree, to the results of the composite WS measures (see Figure A1). The WS index shows highest correlation with GDP per capita and gender equality (both 0.94) (see Table A6). WS factor 1 and WS factor 2 show highest correlation with life expectancy at 65 years (0.96) and social trust (0.89), respectively.

## 4. Discussion

### 4.1. The Association between Welfare State and Mental Wellbeing

“Welfare stateness” is a term used to describe to what degree the state protects and promotes the economic and social wellbeing of its citizens, and it is used here to reflect the strength of the WS based on the measures used in the study. Our results support the hypothesis that welfare stateness is positively associated with MWB within the population. These findings add to the evidence base that shifts focus away from simply identifying the factors influencing MWB, to identifying the conditions under which these factors have an impact [51]. Furthermore, they corroborate previous studies reporting higher levels of happiness and life satisfaction in social democratic WS regimes compared to other regime types [30,33]. Similar results were found by Pacek and Radcliff [52] using panel data for 11 Western European countries covering nearly three decades, years 1975–2002. They found a positive relationship between welfare stateness and life satisfaction using three different measures of WS.

Our study found different levels of associations depending on how WS and MWB were approached. This finding indicates that the relationship observed between WS and MWB is influenced by the way in which these concepts are defined and measured, something which was anticipated given the complexity of both concepts. The current study included two definitions of MWB (i.e., SWB and PWB), which allowed us to explore how differences in definitions can influence WS impact. Although welfare stateness was found to be associated with both SWB and PWB, the impact on the SWB definition was found to be stronger. This confirms the need to consider MWB as a multidimensional construct when exploring its determinants.

Furthermore, our study also illustrates the multidimensional nature of WS. A principal component factor analysis including eight indicators representing different aspects of welfare culture, welfare institutions, policy instruments and outcomes of WS resulted in two separate WS dimensions. The first factor (representing welfare state generosity, gender equality and longevity) was positively associated with both SWB and PWB, while the second factor (representing societal trust, individualism, working-life duration, and income inequality) was only significantly associated with SWB. Approaches using composite WS measures (WS index and WS factors) seem to explain more of the variation in SWB compared to WS regime approach, although this was not found for PWB. This result could indicate that SWB is more related to specific features of WS, as well as policy outputs and outcomes that are captured by the composite WS variables, whereas PWB may be more linked to regional differences captured by the WS regime typology. Separate analyses of each of the eight WS indicators included in WS index and WS factors by and large replicate the results of the composite WS variables in terms of pattern of associations with SWB and PWB, if not effect size. Individually, however, most of them explain less of the between-country variation than the composite WS variables.

Studies of WS regime impact sometimes control for GDP per capita as a variable capturing contextual influence, i.e., level of economic development, besides WS [27,31,33]. Conversely, we have considered national income level as a WS outcome indicator. This alternative approach is supported by the high correlation of GDP per capita and other WS indicators (e.g., the country level variables “social trust” (0.69) and “gender equality” (0.80)). The claim that a strong WS is detrimental to economic growth (based on a hypothesis that a generous WS with high tax burden leads to economic inefficiency) has been contested [53]. How welfare arrangements are designed matters, and the impact on economic growth depends on what taxes are financing. A high share of transfers with strong employment conditionalities (workfare) and larger importance for young and old people, as in the Nordic countries, have been found to support employment and production, balancing concerns for distribution and insurance with economic incentives [53]. Encompassing and universal WS supporting inclusion and equality have been found to contribute to high working life participation and to counteract market failures, e.g., by addressing problems of private health and welfare insurance markets and by providing public goods such as infrastructure, research, education, social security, and social trust, which benefit economic development [32]. Our results do not hinge on the inclusion of national income levels among the WS indicators. The WS index and WS factors are almost perfectly correlated (0.986–0.998) with corresponding WS measures without GDP per capita.

In summary, MWB was associated with WS, although the results do not point to one clear preferable WS model. Different aspects of WS may be more or less important for different aspects of MWB. Rothstein [32] points to several mechanisms by which WS may be linked to SWB, including economic and social equality affecting the subjective health, universal distribution of resources and opportunities supporting social cohesion, and procedural fairness provided by universal access rather than need-testing/means-testing.

### 4.2. The Age Gradient in the Association between Welfare State and Mental Wellbeing

This study explored a potential age gradient in the WS impact on MWB. Our results support the idea that MWB at different ages is linked to circumstances, i.e., that the age gradient in MWB is not uniform, which is reflected by the age patterns of MWB varying with WS levels. We found the direction of the age gradient in MWB to differ depending on the degree of welfare stateness. While a positive or no age gradient was found for certain age groups (from middle aged to old age groups) for high levels of welfare stateness, a negative age gradient (over all ages) was found for low levels of welfare stateness. Hence, we did not find the aforementioned U-shaped age pattern [2] to exist for all countries. Rather, both the magnitude as well as the direction of MWB differences varied by age groups and with WS characteristics.

Our results (based on cross-sectional data) indicated that SWB in countries with high WS scores is consistent with the U-shaped curve for life satisfaction in ages 20 to 70 years as reported by Cheng et al. [19] using longitudinal survey data from western countries. Likewise, our cross-sectional results for the Anglo-Saxon regime, with a mid-life low in the 40s, are in line with results previously reported by Clark [54] using longitudinal survey data from Great Britain covering ages up to 64 years.

A different age pattern emerged for the Eastern regime type, where a clear negative age gradient was found. This result might be explained by a cohort effect, reflecting a so-called “generation gap in inequality aversion” [55] stemming from the older generations being unhappy with developments following the fall of the Communist regime. Alternatively, this effect could reflect a generation gap in prosperity, i.e., a relative deprivation of older generations in comparison to younger generations [56], or that economic hardship hits elderly MWB harder in this regime context [57].

Our results highlight the importance of welfare state policies for MWB, particularly for older adults. WS-related differences in MWB were found to be highest among older adults, pointing to benefits of a “Piggy Bank function” of the WS. Vanhuysse et al. [58] describe European WS systems to be lifecycle redistribution machines and consider age to be a more important determinant for redistribution policy than socio-economic status group. Likewise, Chłoń-Domińczak et al. [59] found that Nordic countries stand out in terms of the age patterns of their public transfers and consumption, having relatively high transfer levels, particularly for the older age group. At the opposite end, they found some of the Eastern European countries (e.g., Bulgaria, Estonia, Latvia, Romania, and Slovenia), which they termed underdeveloped welfare regimes. Generous WS may support adaptation to changing needs, e.g., access to appropriately supportive housing [60]. Access to welfare services and benefits may prolong the period of experienced health and wellbeing into older age, thereby protecting individuals from dependency. Lack of a generous WS regime may have a more significant impact on older adults as they age, and their health care and support needs increase. This could account for the negative age gradient in countries with low values of the WS index, typical for the Eastern regime type, and also those with medium values of the WS index, i.e., the Southern regime type. These are typically countries with family-based welfare and care models [61] and lower alignment between old age-related expenditure and elderly needs [62]. Conde-Sala et al. [63] found poorer quality of life and poorer socioeconomic conditions for the 65+ population living in Eastern and Southern European countries, than in Nordic and Bismarckian countries. They also found education and income levels not to influence quality of life among people aged 65+ in Nordic and Bismarckian countries, as opposed to having a negative impact in Eastern and Southern countries. Hence, WS may compensate for low socioeconomic status in old age.

We also observed a tendency for lower MWB in the oldest old (80+) age group compared to younger old age groups (60–79), even in the context of more developed WS. This result lines up with previous findings implying a decreased ability to (effectively) adapt to changing circumstances in very old age [15]. Bussière et al. [64] studied how MWB adapts to the natural age-related decline in health. They found the health impact on (different measures of) MWB to increase with age, except in the case of life satisfaction when the strength of the relationship decreased with age for individuals under 80 years. However, in the context of the oldest old, health status impact on MWB was also found to increase with age for life satisfaction.

### 4.3. Strengths and Limitations

One strength of the current study is that the multidimensional nature of both WS and MWB has been accounted for. The current study adopted a regime approach to measuring WS, which was supplemented by use of composite measures comprising several aspects of WS culture, institutions, policy instruments and outcomes, representing a flexible approach to capture potential WS effects. Composite indices are still (relatively) rare within empirical analyses of WS [65]. The inclusion of two definitions of MWB (that is, PWB and SWB) broadens our understanding both in terms of the age gradient of MWB, as well as WS impact on MWB. Further research could explore the potential role of WS on other specific dimensions of MWB or specific factors. A further strength of our study is its attention to the oldest old age group. The oldest old are a frequently neglected age group despite deserving more attention, at least in response to the current demographic transition. In particular, our findings relating to the importance of WS in reducing social and health inequality in oldest old age should be followed up in future research.

The following limitations should however be taken into consideration. The cross-sectional nature of the data used in our study means that it is important to acknowledge that any observed age differences could potentially represent cohort or generation effects. For example, one study of longitudinal survey data produced a flat age curve for life satisfaction once cohort effects were controlled for, except for life satisfaction among the oldest old, which was found to reduce sharply with age [66]. Subsequent analyses using longitudinal data are therefore needed in order to shed additional light on the cohort and age effect on MWB as well as their association with WS.

The fact that ESS data are collected from older adults living in the community may lead to our results inadvertently overestimating the MWB of the oldest old age group. Since the likelihood of entering institutional care varies with welfare policy, this could also bias the comparison between WS regimes. Furthermore, the study only includes the adult population. Whether WS is of similar importance to the wellbeing of children and young people should be a research priority in the future.

In the current study, we used data from 2012 in order to capture broader aspects of MWB, which was available from the “Personal and Social Well-Being” module included in the ESS data collection in 2012. Furthermore, as the regime approach has limits due to its static nature, we used different approaches to measure WS, also including the composite index approach as well as showing results for the individual indicators. Both WS measures and their association with MWB at different ages may have changed since then, and the stability of our results should be tested with more recent data.

In a statistical sense, the number of country-level observations included in the multilevel regression model is low. The model included 24 countries, which is below the recommended number of 25 countries for basic linear models [67]. We have approached this by investigating the sensitivity of the results by rerunning the analyses, removing one country at a time. Overall, the results (not shown) are robust to exclusion of individual countries. Most significant results remain for analyses with the WS index. For the WS factors, some weak/borderline results were sensitive to country deletion, such as young age groups for SWB and middle age groups for PWB. For the WS regime results, deletion of Nordic countries (i.e., the reference category), and Finland in particular, affected results for SWB (mostly) for the Bismarckian and Anglo-Saxon regimes. Without Finland, more SWB results in favor of the Nordic regime vis à vis the Bismarckian and Anglo-Saxon were found. An analysis of SWB only for the Nordic countries finds lower SWB for the age groups between 50 and 80 in Finland compared to Denmark, which has the highest average SWB score.

Finally, European countries represent relatively similar social and economic conditions. Studies involving countries with more unequal contexts would shed further light on the connection between WS and MWB and the role of age as a moderator in this relationship.

## 5. Conclusions

Results from the current study indicate that a universal age pattern of MWB does not exist; rather, it seems to be context and policy dependent. The role of the WS is to protect and promote the economic and social wellbeing of its citizens, and this study shows the importance of WS in fostering MWB. Among the adult population studied, the role of WS was found to be particularly important for the MWB of old people. This is a highly important observation considering the increasing number of old people in the coming years. A universal and generous welfare state seems to be particularly important for old people, which often is in high need of transfers and services provided by the WS.

## Figures and Tables

**Table 1:** Sociodemographic characteristics and mental wellbeing of the sample, by regime type. (N = 43,552).

Variables	N	All	Nordic (N = 7722)	Bismarckian (N = 9793)	Anglo-Saxon (N = 4804)	Southern (N = 5944)	Eastern (N = 15,289)
**Sociodemographics**	**N**	**Percentage**					
**Gender**							
Female	23,374	53.7	49.2	51.7	54.7	55.7	56.1
**Age**							
18–29	6917	15.9	17.6	15.3	15.2	15.5	15.8
30–39	6901	15.9	15.4	14.4	17.8	17.4	15.8
40–49	7679	17.6	17.2	18.9	17.5	17.3	17.2
50–59	7804	17.9	17.9	18.5	16.1	16.9	18.5
60–69	7263	16.7	17.3	16.3	16.4	15.7	17.1
70–79	4930	11.3	10.3	11.2	11.2	11.8	11.7
80+	2058	4.7	4.3	5.4	5.9	5.4	3.9
**MWB**	**N**	**Mean (standard deviation)**					
SWB	42,377	3.6 (0.7)	3.9 (0.6)	3.8 (0.6)	3.5 (0.8)	3.4 (0.8)	3.6 (0.7)
PWB	41,152	4.0 (0.5)	4.1 (0.4)	4.1 (0.4)	4.0 (0.5)	4.0 (0.5)	3.9 (0.5)

**Table 2:** Results from multilevel regressions predicting subjective wellbeing (SWB) and psychological wellbeing (PWB) by three welfare state approaches (Model 1, WS index; Model 2, WS factors; Model 3, WS regimes). Model 0, without country-level variables.

	Model 0		Model 1 WS Index		Model 2 WS Factors		Model 3 WS Regimes	
	SWB	PWB	SWB	PWB	SWB	PWB	SWB	PWB
**Gender (ref Male)**								
Female	−0.066 ***	−0.013	−0.06 ***	−0.011	−0.06 ***	−0.011	−0.06 ***	−0.01
**Age-groups (ref 18–29)**							**Ref: Nordic**	
30–39	−0.072 ***	−0.0062	−0.076 ***	−0.0082	−0.076 ***	−0.0076	−0.043	−0.0015
40–49	−0.16 ***	−0.030 *	−0.16 ***	−0.032 **	−0.16 ***	−0.031 **	−0.042 **	0.019
50–59	−0.21 ***	−0.028	−0.22 ***	−0.032 *	−0.22 ***	−0.032 *	−0.012	0.0096
60–69	−0.17 ***	−0.0099	−0.19 ***	−0.02	−0.19 ***	−0.019	0.073	0.043 **
70–79	−0.20 ***	−0.045	−0.22 ***	−0.057 *	−0.22 ***	−0.057 *	0.058	0.034
80+	−0.26 ***	−0.10 *	−0.29 ***	−0.12 ***	−0.30 ***	−0.13 ***	−0.033	−0.073
			**Welfare state and age-interactions**					
			**X1 = WS index**		**X1 = WS factor 1**		**X1 = Bismarckian**	
**X1**			0.099 ***	0.017	0.054 *	0.02	−0.053	−0.022
X1•30–39			0.0082	0.0062	0.0067	0.0071	−0.011	0.00037
X1•40–49			0.074 ***	0.031 **	0.063 ***	0.027 *	−0.04	−0.021
X1•50–59			0.15 ***	0.048 ***	0.11 ***	0.038 **	−0.11 *	−0.0033
X1•60–69			0.22 ***	0.097 ***	0.18 ***	0.099 ***	−0.094	0.035
X1•70–79			0.24 ***	0.12 ***	0.20 ***	0.12 ***	−0.092	0.025
X1•80+			0.22 ***	0.13 ***	0.18 ***	0.15 ***	−0.15 **	0.069
					**X2 = WS factor 2**		**X2 = Anglo-Saxon**	
**X2**					0.087 ***	0.00094	−0.18 ***	−0.13 ***
X2•30–39					0.0051	0.0028	−0.047	0.018
X2•40–49					0.042 *	0.018	−0.16 ***	−0.062 **
X2•50–59					0.11 ***	0.030*	−0.072	0.054
X2•60–69					0.14 ***	0.037	−0.014	0.13 ***
X2•70–79					0.15 ***	0.053	−0.02	0.12 ***
X2•80+					0.14 ***	0.044	0.11 **	0.25 ***
							**X3 = Southern**	
**X3**							−0.20 ***	−0.0097
X3•30–39							−0.081 *	−0.015
X3•40–49							−0.16 ***	−0.052
X3•50–59							−0.28 ***	−0.066
X3•60–69							−0.35 ***	−0.065
X3•70–79							−0.33 **	−0.095
X3•80+							−0.32 *	−0.045
							**X4 = Eastern**	
**X4**							−0.19 **	−0.064
X4•30–39							−0.035	−0.017
X4•40–49							−0.18 ***	−0.083 **
X4•50–59							−0.36 ***	−0.098 ***
X4•60–69							−0.51 ***	−0.19 ***
X4•70–79							−0.55 ***	−0.24 ***
X4•80+							−0.50 ***	−0.23 ***
Constant	3.82 ***	4.06 ***	3.80 ***	4.05 ***	3.80 ***	4.05 ***	3.94 ***	4.10 ***
ICC (se)	0.101 (0.022)	0.045 (0.013)	0.031 (0.007)	0.03 (0.007)	0.028 (0.008)	0.029 (0.007)	0.042 (0.01)	0.027 (0.006)
R^2^ country-level (SB/BR)			0.72/0.72	0.33/0.34	0.75/0.75	0.37/0.38	0.62/0.62	0.41/0.41
AIC	87,278.25	53,137.62	86,575.33	52,818.1	86,566.72	52,757.2	86,554.9	52,691.87
BIC	87,364.8	53,223.87	86,722.46	52,964.73	86,774.42	52,964.2	86,883.76	53,019.62
N			42,377	41,152	42,377	41,152	42,377	41,152

Twenty-four countries at the country level. *** *p* < 0.001, ** *p* < 0.01, * *p* < 0.05. ICC = Intra class correlation, se, standard error; SB, Snijders/Bosker; BR, Bryk/Raudenbush; AIC, Akaike information criterion; BIC, Bayesian information criterion.

## Data Availability

Raw data are from the European Social Survey (ESS) and from other open sources, i.e,. Eurostat, the World Bank DataBank, ESS Multilevel Data Repository and Hofstede Insights. The European Social Survey data are available to download after registration at https://www.europeansocialsurvey.org/data/download.html?r=6 (accessed on 17 January 2018). The ESS ERIC, Core Scientific Team (CST), and the producers bear no responsibility for the uses of the ESS data or for interpretations or inferences based on these uses. The responsibility for all conclusions drawn from the data lies entirely with the authors.

## Appendix A

### Welfare State Approaches

The literature on how WS impacts health and wellbeing is inconclusive [68,69]. WS is a broad concept, making it less likely for empirical findings to converge. Different parts of WS may matter for different health-related outcomes, and empirical findings may also vary in relation to how WS is defined.

Bergquist et al. [70] identifies three approaches for comparative research of WS, the “regime approach”, the “institutional approach” and the “expenditure approach”. Kunißen [65] also defines three strategies for operationalizing WS, the “the regime approach”, “the composite index approach” and “the single indicator approach”. As can be expected, the approaches identified by Bergquist et al. and Kunißen (partially) overlap. Most studies on health effects have used the “regime approach” [70].

Studies employing the “regime approach” typically depart from the research tradition initiated by the Three Worlds of Welfare Capitalism [42], which classified countries into Social Democratic, Conservative and Liberal regime types. Social Democratic regimes are characterized by a comprehensive system of social protection with generous benefits based on citizenship (right-based universalism) and upholding public provision of services. These regimes ensure that livelihoods can be maintained without reliance on the market (decommodification). Conservative regimes, however, are characterized by “Bismarkian” systems of worker insurance and company-based social protection schemes, with cash-based social transfers related to earnings, i.e., rights and benefits are attached to class and status. This approach is based on the principle of subsidiarity, that is, that the state will only interfere when the family’s capacity to service its members is exhausted ([42], p. 29). Although conservative regimes can be generous, they rely on group-based solidarity, with negligible redistribution between social groups, which contrasts with right-based universalism. Liberal regimes target their welfare policy toward the poor, with modest, means-tested benefits, modest universal transfers, and modest social insurance minimizing decommodification. The development of the Esping Andersen regime typology was based on a limited set of countries, with later (European) adoptions adding Mediterranean and Post-Communist regime types. Mediterranean regimes are characterized by a highly fragmented welfare system and high reliance on the family and the voluntary sector [71,72]. Post-Communist regimes, however, are characterized by a high take-up of social security, with the same program covering everyone, albeit with relatively low benefit levels. Furthermore, Post-Communist regimes are characterized by low levels of state institution trust, and standards of living being largely determined by the market and the family [73]. General features of regime types adapted from Chung et al. [74] are summarized in Table A1.

**Table A1:** General features of welfare state regimes ^a^.

Regime	Population Coverage ^b^	Role of the Private Market ^c^	Target Population ^d^	Decommodification ^e^
Social Democratic	Universal	Low	All citizens	High
Conservative	Occupational	Low	Families	Medium
Liberal	Selective	High	Poor	Low
Mediterranean	Occupational	Medium	Families	Low
Post-Communist	Selective	Medium	Poor	Low

(^a^) Adapted from Chung et al. [74]. (^b^) Population Coverage, share of the population eligible or covered for welfare services and benefits; (^c^) Role of Private Market, degree to which welfare needs are met through the private sector; (^d^) Target Population, individuals or groups that are identified as the primary and intended recipients of welfare services and benefits; (^e^) Decommodification, degree to which welfare benefits reduce individuals’ reliance on the market to meet basic needs.

Esping Andersen based his classification of WS regimes on developments of WS in a historical–political–geographical context [75]. Rice [43] reaches a similar four-type classification of (western) European countries based on an ideal-type conceptual model. This classification builds on a three-dimensional model (welfare culture, welfare institutions and socio-structural effects) where each dimension is organized into two juxtaposed axes: conservatism vs. liberalism (or socially conservative vs. socially transformative) and solidarism vs. residualism (or economically conservative vs. economically transformative). This gives way to the four ideal types of WS regimes, i.e.,: Socio-liberal regime (Nordic), Liberal regime (Anglo-Saxon), Socio-conservative regime (Continental) and Conservative regime (Southern).

The best approach for classifying countries according to regime types remains unclear. Esping Andersen’s classification has, for example, only received partial support within empirical literature [45,76,77,78]. A general critique of using the “Regime approach” in empirical applications has been that regime typologies describe ideal types that individual countries comply with to varying degrees [46,79]. A further area of disagreement concerns the multitude of regime types and inconsistency in the grouping of countries in line with these [45,46,80,81]. Notwithstanding these issues, different countries may have some common features that may be important for some (even if not all) aspects of population health and wellbeing.

An approach that relies solely on classification based on regime typology may prove to be too rigid and may fail to capture potential variation within regime types. Erroneous classification of individual countries also has the potential to mask any regime differences. Consequently, using approaches that build on the measurement of specific policy instruments, or outcomes of welfare regimes, may provide a deeper understanding of WS impact on health and wellbeing [82]. For this reason, we have opted to include both the regime approach and the composite index approach to measure “welfare stateness” in this context [65].

## Appendix B

**Table A2:** Variables used in factor analyses of welfare state.

Variable	Description
GDP per capita	Gross Domestic Product per capita in PPS, 2012, Index (EU28 = 100), Eurostat
Health/GDP + Social/GDP	Health expenditure as % of GDP, the World Bank DataBank, plus Social protection expenditure as % of GDP, 2012, Eurostat
Gender empowerment measure	Human Development Report 2009, United Nations (compiled by the ESS Multilevel Data Repository)
Life expectancy at 65	Life expectancy in absolute value at 65 (average for male and female), 2012, Eurostat
Duration working life	Duration of working life, 2012, Eurostat
GINI	Gini coefficient of equivalized disposable income (scale from 0 to 100), EU-SILC survey, 2012, Eurostat
Individualism	Individualism versus Collectivism Index, from Hofstede Insights, https://www.hofstede-insights.com/product/compare-countries/ (accessed on 28 May 2019). Description: The high side of this dimension, called individualism, can be defined as a preference for a loosely knit social framework in which individuals are expected to take care of only themselves and their immediate families. Its opposite, collectivism, represents a preference for a tightly knit framework in society in which individuals can expect their relatives or members of a particular ingroup to look after them in exchange for unquestioning loyalty. A society’s position on this dimension is reflected in whether people’s self-image is defined in terms of “I” or “we”.
Trust	Average rating of trust by domain (trust police, trust legal system, trust political system, trust others), both sexes, all educational attainment levels, 16 years or over (rating 0–10), EU—SILC survey, 2013, Eurostat, http://appsso.eurostat.ec.europa.eu/nui/show.do?dataset=ilc_pw03 (accessed on 28 May 2019).

**Table A3:** Items included in the calculation of mental wellbeing measures ^a^.

Mental Wellbeing Factor	Items
*Subjective wellbeing (SWB)*	
Evaluative wellbeing	How satisfied with life as a whole, How happy are you
Positive emotional wellbeing	Enjoyed life, Were happy, Felt calm and peaceful, Had lot of energy (how often past week)
*Psychological wellbeing (PWB)*	
Positive functioning	Free to decide how to live my life, Feel accomplishment from what I do, Feel what I do in life is valuable and worthwhile, There are lots of things I am good at, In general feel very positive about myself, Always optimistic about my future
Flow	Interested in what you are doing, Absorbed in what you are doing, Enthusiastic about what you are doing
Positive relationships	Feel people treat you with respect, Feel appreciated by people you are close to, Provide help and support to people you are close to, Receive help and support from people you are close to

(^a^) Only items with factor loadings >0.30 in two of the three age groups used in the original ESEM-analysis were retained.

**Table A4:** Results of factor analyses of welfare state variables.

Variable	Restricting to One Factor		Using the Kaiser Criterion		
	Factor1	Uniqueness	Factor1 ^a^	Factor2 ^a^	Uniqueness
GDP per capita	0.9191	0.1552	***0.6552***	***0.6583***	0.1374
Health/GDP + Social/GDP	0.7738	0.4012	**0.9023**	0.1834	0.1522
Gender empowerment measure	0.9178	0.1576	**0.7611**	0.5421	0.1269
Life expectancy at 65	0.7532	0.4327	**0.9568**	0.0869	0.077
Work life duration	0.7285	0.4692	0.4464	**0.** **6272**	0.4073
GINI	−0.5459	0.702	−0.1759	**−0.** **6969**	0.4834
Individualism	0.5364	0.7123	0.2092	**0.6422**	0.5438
Trust	0.7124	0.4924	0.1747	**0.8928**	0.1723

(^a^) After orthogonal rotation of the axes. Bold indicates factor loading >0.60. Italic indicates equal loading.

**Table A5:** Country-level R-squared (Snijders/Bosker /Bryk/Raudenbush).

WS-Variable	SWB	PWB
WS index	0.72/0.72	0.34/0.34
WS factors	0.75/0.75	0.37/0.38
WS regimes	0.62/0.62	0.41/0.42
GDP per capita	0.68/0.67	0.28/0.28
Health and social spending (% of GDP)	0.33/0.33	0.25/0.26
Gender equality	0.54/0.54	0.27/0.28
Longevity at 65	0.41/0.41	0.33/0.34
Working life duration	0.55/0.55	0.24/0.24
Gini coefficient	0.42/0.42	0.00/0.00
Individualism	0.12/0.12	0.01/0.01
Social trust	0.51/0.52	0.19/0.19

**Table A6:** Correlation matrix of the WS index and two WS factors and included indicators.

	WS Index	WS Factor 1	WS Factor 2	GDP	Health and Social Spending	Gender Equality	Life Expectancy at 65	Working Life Duration	Gini	Individualism	Social Trust
**WS index**	1										
**WS factor 1**	0.7555	1									
**WS factor 2**	0.648	0	1								
**GDP**	0.9404	0.6552	0.6583	1							
**Health and Social spending**	0.7917	0.9023	0.1834	0.6675	1						
**Gender equality**	0.939	0.7611	0.5421	0.8022	0.7794	1					
**Life expectancy at 65**	0.7706	0.9567	0.087	0.6986	0.8113	0.7241	1				
**Working life duration**	0.7454	0.4464	0.6273	0.6785	0.3657	0.6788	0.5224	1			
**Gini**	−0.559	−0.176	−0.697	−0.579	−0.339	−0.471	−0.239	−0.411	1		
**Individualism**	0.5488	0.2093	0.6421	0.5102	0.3886	0.5168	0.2411	0.2638	−0.297	1	
**Social trust**	0.7289	0.1747	0.8928	0.6915	0.3406	0.6112	0.2461	0.6484	−0.493	0.544	1
